# ECG Predictors for New-Onset Atrial Fibrillation Within a Year After Radiofrequency Ablation of Counterclockwise-Rotating Atrial Flutter

**DOI:** 10.3389/fcvm.2021.739350

**Published:** 2021-11-12

**Authors:** Hailei Liu, Zhoushan Gu, Chao Zhu, Mingfang Li, Jincheng Jiao, Hongwu Chen, Gang Yang, Weizhu Ju, Kai Gu, Fengxiang Zhang, Lin Yee Chen, Di Yang, Minglong Chen

**Affiliations:** ^1^Division of Cardiology, The First Affiliated Hospital of Nanjing Medical University, Nanjing, China; ^2^Cardiovascular Division, Department of Medicine, University of Minnesota Medical School, Minneapolis, MN, United States

**Keywords:** typical AFL, new-onset AF, ECG components, predicting, ablation

## Abstract

**Background:** New-onset atrial fibrillation (AF) after ablation of typical atrial flutter (AFL) is not rare. This study aimed to investigate the predictive value of electrocardiographic parameters on new-onset AF post-typical AFL ablation.

**Methods:** A total of 158 consecutive patients (79.1% males, mean age 57.8 ± 14.3 years) with typical AFL were enrolled between January 2012 and August 2017 in this single-center study. Patients with a history of AF before ablation were excluded. ECGs during sinus rhythm (SR) and AFL were collected. The duration of the negative component of flutter wave in lead II (D_FNII_), proportion of the D_FNII_ of the total circle length of AFL (D_FNII_%), amplitude of the negative component of flutter wave in lead II (A_FNII_), duration (D_PNV1_), and amplitude (A_PNV1_) of negative component of the P wave in lead V1, and P wave duration in lead II (D_PII_) during sinus rhythm were measured.

**Results:** During a median follow-up of 26.9 ± 11.8 months, 22 cases (13.9%) developed new-onset AF. D_FNII_ was significantly longer in patients with new-onset AF compared to patients without AF (114.7 ± 29.6 ms vs. 82.7 ± 12.8 ms, *p* < 0.0001). A_FNII_ was significantly lower (0.118 ± 0.034 mV vs. 0.168 ± 0.051 mV, *p* < 0.0001), D_PII_ (144.21 ± 23.77 ms vs. 111.46 ± 14.19 ms, *p* < 0.0001), and D_PNV1_ was significantly longer (81.07 ± 16.87 ms vs. 59.86 ± 14.42 ms, *p* < 0.0001) in patients with new-onset AF. In the multivariate analysis, D_FNII_ [odds ratio (OR), 1.428; 95% CI, 1.039–1.962; *p* = 0.028] and D_PII_ (OR, 1.429; 95% CI, 1.046–1.953; *p* = 0.025) were found to be independently associated with new-onset AF after typical AFL ablation.

**Conclusion:** Parameters representing left atrial activation time under both the SR and AFL were independently associated with new-onset AF post-typical AFL ablation and may be useful in risk prediction, which needs to be confirmed by further prospective studies.

## What Is Known?

- New-onset AF after ablation of typical AFL is not rare.
- Some ECG parameters such as P wave duration and amplitude and fragmented QRS complex under SR are predicators of AF after AFL ablation.

## What the Study Adds?

- Electrocardiogram parameters during both the AFL and SR were useful in predicting the risk of new-onset AF in typical patients with AFL post-ablation.
- Duration-related parameters such as duration of the negative component of flutter wave in lead II (D_FNII_) and P wave duration in lead II during SR (D_PII_) were independent risk predictors of the occurrence of AF.

## Introduction

Typical atrial flutter (AFL) is a frequently encountered clinical tachyarrhythmia, especially occurring in elderly people. Given the high success rate and low risk, radiofrequency catheter ablation (RFCA) is currently the first-line treatment of AFL. However, previous studies showed that even in patients without previously documented atrial fibrillation (AF), the risk of new-onset AF after AFL ablation was relatively high (1–4), rendering either a second AF ablation or a delayed AF management. Therefore, it is of great significance to screen such patients.

Currently, predictors for new-onset AF in patients with AFL are mainly based on baseline clinical characteristics including body mass index (BMI) (5), obesity (6), diabetes (7), heart failure (8), chronic renal insufficiency (9), and the Congestive heart failure/left ventricular ejection fraction ≤40%, Hypertension, Age ≥75 years, Diabetes mellitus, Stroke/transient ischemic attack/thromboembolism history, Vascular disease, Age 65–74 years, Sex (female) (CHA_2_DS_2_-VASc) score (10). Other clinical characteristics such as left atrial volume (11), ECG parameters (12, 13), left atrial diameter (LAD) (14), and left ventricular ejection fraction (LVEF) (15) were also reported to be useful. Since the biatrial activation and wavefront conduction patterns during both the AFL and sinus rhythm (SR) were studied (16–18), the relationship between the regional activation and the corresponding components of surface ECG had been well-established (16). Therefore, the disease of the atria can be reflected by the changes of these components. We, thus, hypothesized that such changes are more intuitive and direct for predicting AF occurrence post-AFL ablation because AF itself is the clinical presentation of atrial cardiomyopathy (19).

Thus, ECG parameters, which could be easily obtained, were widely used for predicting new-onset AF after AFL ablation. Currently, P wave duration (20) and amplitude (21, 22) under SR are more commonly used. However, different rhythms have different blind areas when projecting to surface ECG. The components of P wave were sometimes difficult to measure, especially in patients with small P wave. Using the components of a more prominent wave—flutter wave under AFL to predict the post-procedural AF occurrence has not been reported. In this study, we investigate the predictive value of electrocardiographic parameters obtained from P wave and flutter wave on new-onset AF post-typical AFL ablation.

## Methods

### Patients

From January 2012 to August 2017, patients with typical counterclockwise-AFL (CCC-AFL) referring for ablation in the Nanjing Medical University Hospital were consecutively enrolled. The ECG definition of typical AFL (CCW-AFL) was previously reported (16). Exclusion criteria included: (1) Documented AF episodes before ablation; (2) Contraindication to ablation; (3) Life expectancy <1 year; and (4) Clockwise AFL was also excluded, since its proportion was very small. After admission, routine tests and echocardiogram were performed. Transesophageal echocardiogram or CT was conducted to rule out atrial thrombosis. Written informed consent was obtained before operation. The clinical protocol was approved by the ethics committee.

### Electrocardiogram Measurement

**Figure 1:**
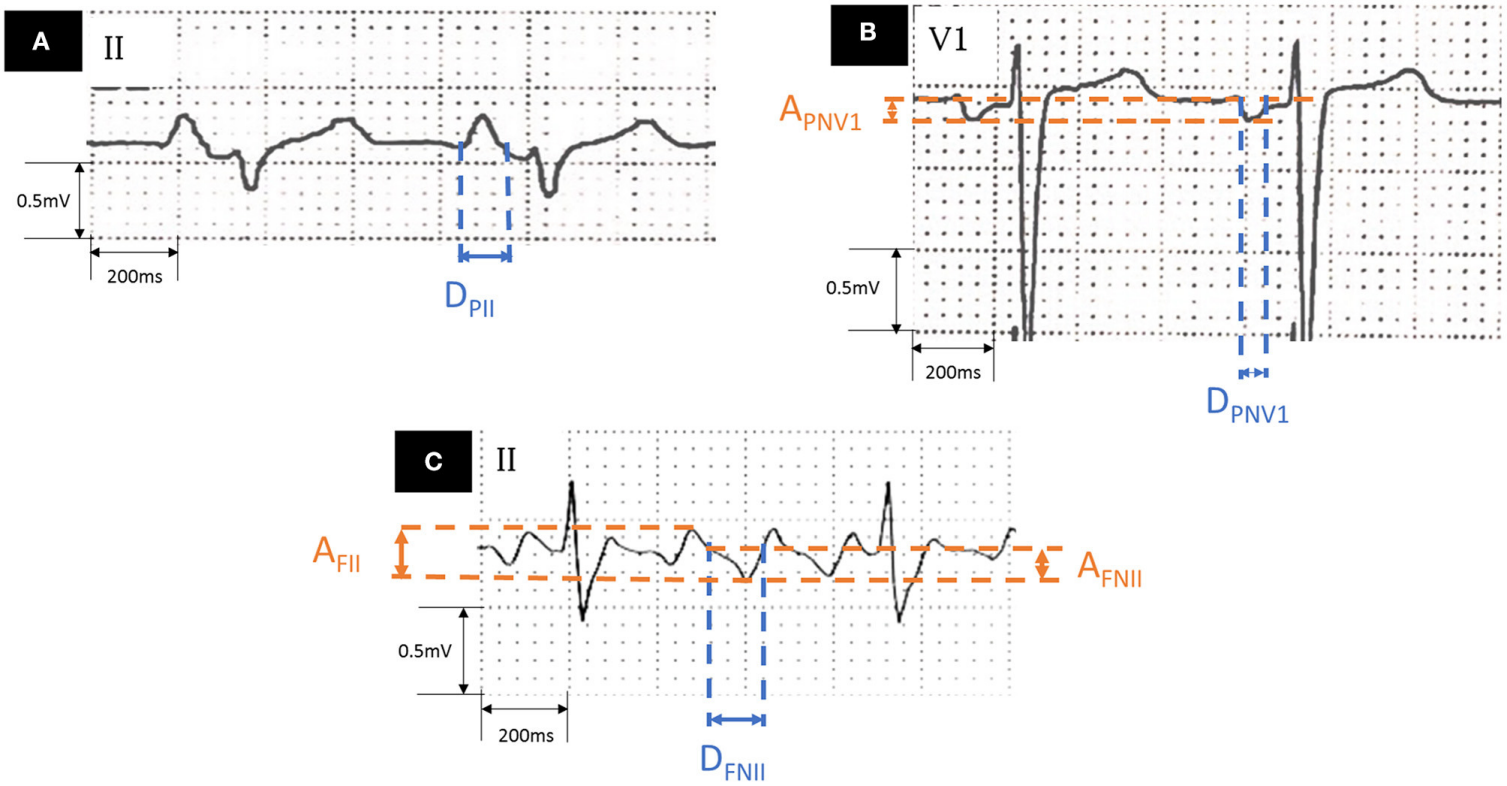
Measurement of ECG parameters. **(A)** Lead II during sinus rhythm. **(B)** Lead V1 during sinus rhythm. **(C)** Lead II during AFL. AFL, atrial flutter; A_FNII_, amplitude of the negative component of flutter wave in lead II; A_PNV1_, amplitude of negative component of the P wave in lead V1 during sinus rhythm; D_FNII_, duration of the negative component of flutter wave in lead II; D_PII_, P wave duration in lead II during sinus rhythm; D_PNV1_, duration of negative component of the P wave in lead V1 during sinus rhythm.

Standard 12-lead ECGs during CCW-AFL and SR were obtained (filter range 0.5–100 Hz; AC filter 50 Hz, 25 mm/s, 10 mm/mV) before procedure. Antiarrhythmic drugs (AADs) were discontinued for at least 5 half-lives before ECG collection. If ECG during SR or AFL could not be obtained after enrollment, the patients were asked to provide the most recent ECG free from any AADs influence (administration of AADs within 1 month before the ECG acquisition). An obvious deflection point between each flutter wave was treated as the baseline according to a previous study (16). The ECG component that reflects left atrial activation during CCW-AFL or SR was measured as follows (Figure 1): (1) duration of the negative component of flutter wave in lead II (D_FNII_); (2) the proportion of D_FNII_ of total CCW-AFL circle length (D_FNII_ %); (3) the amplitude of the negative component of flutter wave in lead II (A_FNII_); (4) the total amplitude of the flutter wave in lead II (A_FII_); (5) the amplitude of negative component of P wave in lead V1 during SR (A_PNV1_); (6) the duration of negative component of P wave in lead V1 during SR (D_PNV1_); and (7) duration of P wave in lead II during SR (D_PII_). P wave terminal force of V1 (PtfV1) was also calculated. Adobe Acrobat XI Pro (Adobe, California, USA) was used to do the measurement by two independent experienced cardiologists who were not involved in this study. The value provided by each cardiologist was an average value of three different beats. The final value was the average of the results by each cardiologist. The values would be remeasured to reach a consensus by both the cardiologists under at least one the following circumstances to control the interobserver variation: (1) The amplitude difference under SR was over 0.05 mV; (2) The amplitude difference under AFL was over 0.1 mV; (3) The duration difference under sinus rhythm was over 0.04 s; and (4) The duration difference under AFL was over 0.08 s.

### Radiofrequency Catheter Ablation

Antiarrhythmic drugs were discontinued for at least 5 half-lives before procedure, except the ß-blockers for rate control. Electrophysiological study was performed under local anesthesia. Spontaneous or induced CCW-AFL was recorded. Activation mapping was performed by using a three-dimensional mapping system [Carto system (Biosense-Webster, Diamond Bar, CA) or EnSite NavX system (Abbott, Abbott Park, IL)] and cavotricuspid isthmus-dependent CCW-AFL was confirmed with entrainment mapping. Under the guidance of the three-dimensional mapping system, an irrigated catheter was used to perform linear ablation between the tricuspid annulus and the inferior vena cava. Bidirectional block across the line was adopted as ablation endpoint.

### Follow-Up of Patients

No AADs were prescribed after the procedure. Patients were regularly followed every 3 months with ECG and 24-h Holter recordings within the first year. ECGs were obtained when patients experienced any palpitation within the first year and thereafter. CCW-AFL recurrence was defined as AFL longer than 30 s during follow-up after 3 months of blanking period. New-onset AF was defined as any episode of AF lasting over 30 s during follow-up period.

### Statistical Analysis

Data were expressed as mean ± SD for continuous variables and as absolute frequency and percentage for categorical variables. All the statistical analysis was done by using the Statistical Package for the Social Sciences (SPSS) version 20.0 (International Business Machines Corporation, Armonk, NY) and the R software version 4.0.3 (The R Foundation for Statistical Computing, Vienna, Austria). The differences between groups were compared by using the Student's *t*-test or the chi-squared test. Two-sided *p* < 0.05 was considered as statistical significant. To identify factors associated with new-onset AF post-AFL ablation, the multivariate logistic regression was performed (forward LR method). The DeLong's test was used to compare C-statistic between models and the net reclassification improvement and integrated discrimination improvement were done to compare reclassification.

## Results

### Baseline Characteristics and Follow-Up Results

We enrolled 158 patients (mean age 57.8 ± 14.3 years, 78.9% males) in this study. Ablation endpoint was achieved in all the patients. During a mean follow-up period of 26.9 ± 11.8 months, six patients (3.8%) had AFL recurrence and need a second ablation. Among all the patients, 22 patients (13.9%) developed new-onset AF. Mean duration of AF seen on Holter was 213 ± 48 s, 19 patients had symptomatic AF and 17 patients needed AADs or ablation for AF. All the patients were divided into two groups according to new-onset AF. The baseline characteristics were shown in Table 1 and no significant differences were found between the two groups regarding age, gender, LAD, LVEF, the CHA_2_DS_2_-VASc score, PR interval, and the HATCH score (Table 1).

**Table 1:** Comparison of the parameters between patients with and without atrial fibrillation.

	**Non-AF (*n* = 136)**	**AF (*n* = 22)**	***p*-value**
Age(y)	57.4 ± 14.8	59.9 ± 10.7	0.347
Male (%)	107 (78.7)	17 (77.3)	0.882
**Comorbidities**			
Hypertension (%)	60 (44.1)	11 (50)	0.607
Diabetes mellitus (%)	39 (28.7)	7 (31.8)	0.763
Congestive heart failure (%)	21 (15.4)	3 (13.6)	1.000
Structural heart disease (%)	16 (11.8)	3 (13.6)	0.731
CHA_2_DS_2_-VASc	1.64 ± 1.17	1.64 ± 1.22	0.990
HAS-BLED	1.82 ± 0.75	1.95 ± 0.90	0.500
HATCH score	0.97 ± 1.01	0.95 ± 1.05	0.947
**Echocardiographic parameters**			
LAD (mm)	39.0 ± 5.6	39.6 ± 5.9	0.631
LVEF (%)	61.6 ± 7.6	61.5 ± 7.9	0.974
**Electrocardiographic parameters**			
PR interval (ms)	181.68 ± 39.59	184.38 ± 45.60	0.800
D_FNII_ (ms)	82.30 ± 12.39	121.52 ± 22.63	<0.001
TCL(ms)	211.17 ± 23.05	215.68 ± 23.61	0.447
A_FNII_ (mV)	0.16 ± 0.06	0.11 ± 0.05	<0.001
A_FII_ (mV)	0.26 ± 0.08	0.26 ± 0.07	0.795
A_PNV1_ (mV)	0.06 ± 0.05	0.06 ± 0.04	0.077
D_PII_ (ms)	112.28 ± 6.89	125.18 ± 8.61	<0.001
D_PNV1_(ms)	31.25 ± 31.75	51.59 ± 42.06	<0.001
PtfV1(mm*ms)	−3.33 ± 3.89	−4.54 ± 4.24	0.406

*Values are presented as mean ± SD or n (%). AFL, atrial flutter; A_FNII_, amplitude of the negative component of flutter wave in lead II; A_PNV1_, amplitude of negative component of the P wave in lead V1 during sinus rhythm; D_FNII_, duration of the negative component of flutter wave in lead II; D_PII_, P wave duration in lead II during sinus rhythm; D_PNV1_, duration of negative component of the P wave in lead V1 during sinus rhythm, LAD, left atrial diameter; LVEF, left ventricular ejection fraction; PtfV1, P terminal force of V1*.

### Electrocardiogram Parameters

**Figure 2:**
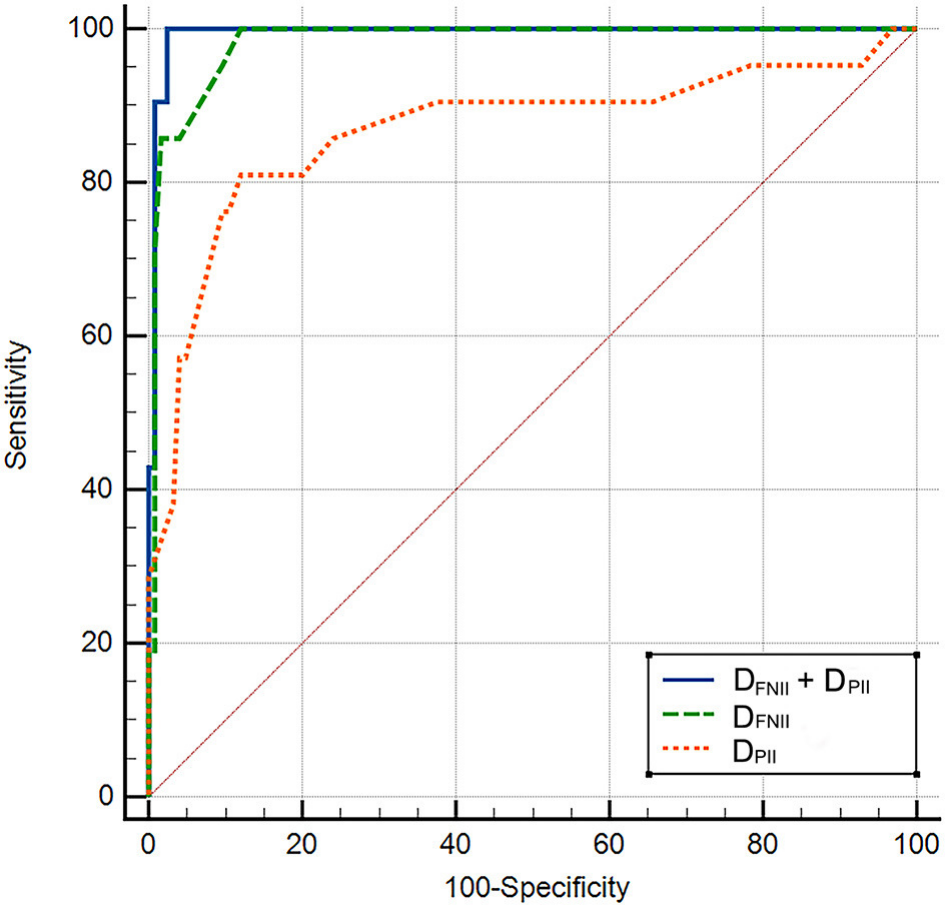
The receiver operating curve for each model. D_FNII_, duration of the negative component of flutter wave in lead II; D_PII_, P wave duration in lead II during sinus rhythm.

Compared to patients without new-onset AF, D_FNII_ (114.7 ± 29.6 ms vs. 82.7 ± 12.8 ms, *p* < 0.0001), D_PII_ (144.21 ± 23.77 ms vs. 111.46 ± 14.19 ms, *p* < 0.0001), and D_PNV1_ (81.07 ± 16.87 ms vs. 59.86 ± 14.42 ms, *p* < 0.0001) were significantly prolonged in patients with new-onset AF. A_FNII_ was significantly reduced (0.118 ± 0.034 mV vs. 0.168 ± 0.051 mV, *p* < 0.0001) in patients with new-onset AF. No significant difference was found between two groups regarding A_PNV1_ (0.086 ± 0.022 mV vs. 0.103 ± 0.034 mV, *p* = 0.077). D_FNII_, A_FNII_, D_PNV1_, D_PII_, the CHA_2_DS_2_-VASc score, and LAD were adjusted in the multivariate regression analysis and D_FNII_ and D_PII_ were independent predictors of new-onset AF after CCW-AFL ablation (Table 2). The C-statistic for D_FNII_, D_PII_, and D_FNII_ + D_PII_ and the differences of predictive performances between each model were shown in Table 3. The receiver operating curve for each model was shown in Figure 2.

**Table 2:** The univariate and multivariate Cox regression analysis for identifying predictors of new-onset atrial fibrillation.

	**Univariate analysis**			**Multivariate analysis**		
	**OR**	**95% CI**	***p*-value**	**OR**	**95% CI**	***p*-value**
D_FNII_	1.248	1.133–1.375	**<0.001**	1.428	1.039–1.962	**0.028**
A_FNII_	0.001	0.00–0.001	**<0.001**	0.384	0.000–2.160	0.965
D_PNV1_	1.018	1.004–1.032	**0.012**	1.029	0.971–1.091	0.331
D_PII_	1.265	1.155–1.385	**<0.001**	1.429	1.046–1.953	**0.025**
CHA_2_DS_2_-VASc	0.998	0.678–1.469	0.990	0.753	0.099–5.713	0.784
LAD	1.022	0.940–1.112	0.610	1.080	0.745–1.566	0.685

*A_FNII_, amplitude of the negative component of flutter wave in lead II; D_FNII_, duration of the negative component of flutter wave in lead II; D_PII_, P wave duration in lead II during sinus rhythm; D_FNII_%, proportion of the D_FNII_ of the total circle length of atrial flutter; D_PNV1_, duration of negative component of the P wave in lead V1 during sinus rhythm; LAD, left atrial diameter; OR, odds ratio. Bold values represent statistical significance*.

**Table 3:** Differences of predictive performances of each model.

**Model**	**C statistic**	**95% CI**	**Z-score**	***p*-value**	**NRI**	**Z-score**	***P*-value**	**IDI**	**Z-score**	***p*-value**
D_PII_	0.868	0.777-0.979	–	–	–	–	–	–	–	–
D_FNII_	0.982	0.964-1.000	1.998	0.046	0.136	1.261	0.207	0.122	1.073	0.283
D_FNII_+D_PII_	0.994	0.982-1.000	2.295	0.021	0.148	1.399	0.162	0.431	3.256	0.001

*D_FNII_, duration of the negative component of flutter wave in lead II; D_PII_, P wave duration in lead II during sinus rhythm; IDI, integrated discrimination improvement; NRI, net reclassification improvement*.

## Discussion

The results of this study demonstrated that D_FNII_ and D_PII_ were independent predictors of new-onset AF after CCW-AFL ablation.

### Relationship Between ECG Components During CCW-AFL and Their Corresponding Activation of the Atria

**Figure 3:**
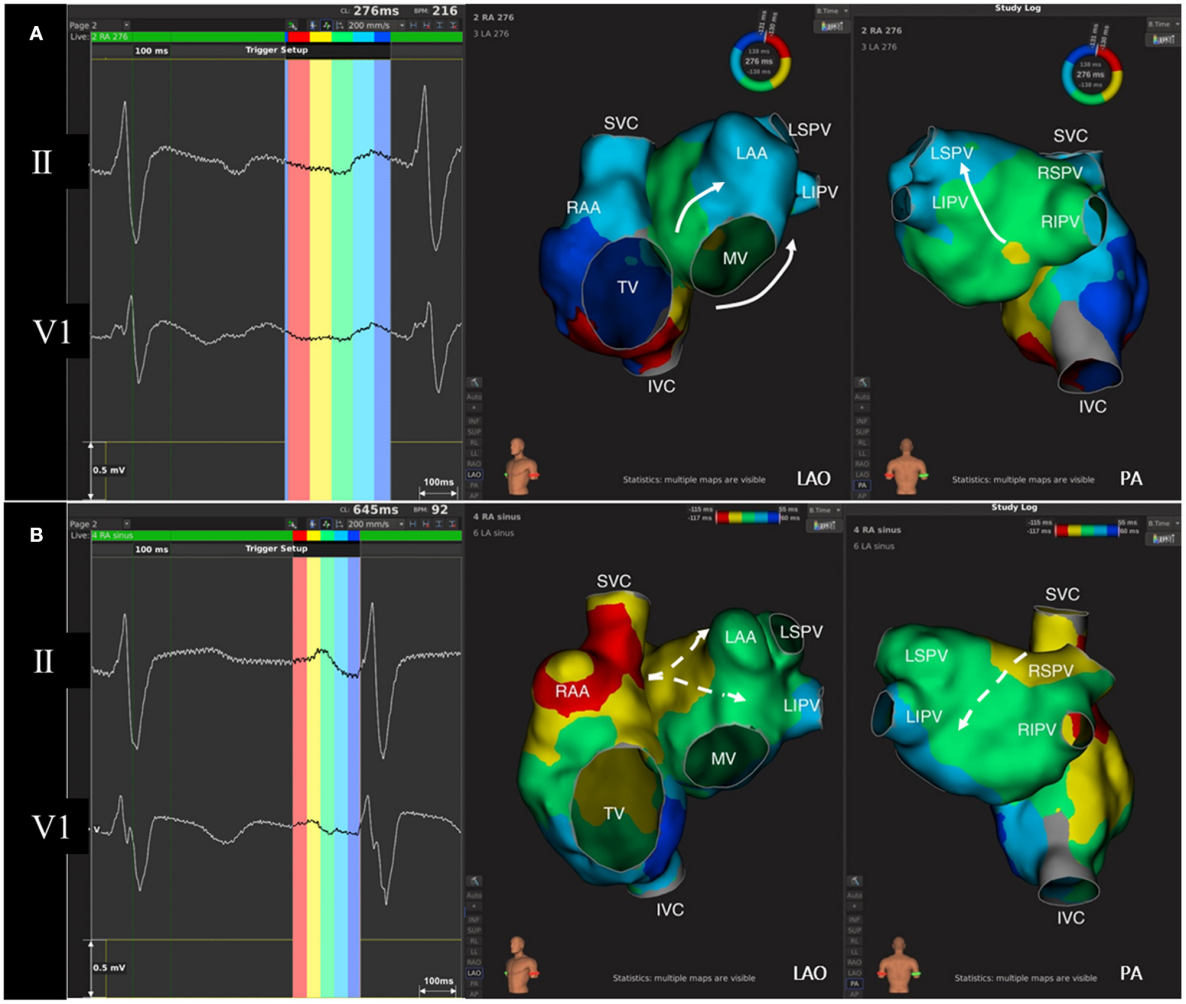
The correlation between regional atrial activation and the components of flutter wave and P wave. **(A)** Flutter wave and isochronal activation mapping of both the atria during AFL. Left atrium activation during typical counterclockwise AFL goes from inferior-septal wall to superior-lateral wall (white arrow). The negative component of flutter wave in lead II corresponds to the activation of right atrial septum and left atrium, while the positive component of flutter wave indicates right atrial free-wall activation. **(B)** P wave and isochronal activation mapping of both the atria during sinus rhythm. P wave in lead V1 is normally biphasic. The initial positive force indicates right atrial activation, while left atrial activation from septum to free wall (white dashed arrow) contributes to the terminal negative force in lead V1. AFL, atrial flutter; IVC, inferior vena cava; LAA, left atrial appendage; LAO, left anterior oblique; LIPV, left inferior pulmonary vein; LSPV, left superior pulmonary vein; MV, mitral valve; PA, posterior-anterior; RAA, right atrial appendage; RIPV, right inferior pulmonary vein; RSPV, right superior pulmonary vein; SVC, superior vena cava; TV, tricuspid valve.

As shown in Figure 3, the relationship between ECG components during SR and their corresponding activation of the atria has been well-established. Recently, with the three-dimensional electroanatomic mapping, such a match has drawn the attention of clinical electrophysiologists. Using ultra-high density mapping technique (Figure 3), the activation and propagation pattern of both the atria during CCW-AFL could also be clearly manifested. Cava-tricuspid isthmus conduction and the lower septal activation are projected onto the slow downslope of the flutter wave in the inferior leads, while the activation of the upper septum and the whole left atrium was reflected as their negative component. The terminal positive component of the inferior leads during CCW-AFL represents the activation of the right atrial free wall coming from the upper to lower. Other previous studies had also demonstrated the similar findings (16, 17).

### Atrial Fibrosis and AF

Given the complex pathophysiological process, the etiology of AF is not completely understood at present. Haissaguerre found that the occurrence and maintenance of AF were related to the triggering foci, which mainly originated from pulmonary veins (23). Thereafter, pulmonary vein isolation (PVI) has become the cornerstone for ablation of AF. However, despite a variety of ablation tools and strategies that were developed in the past few decades, the success rate of persistent AF ablation was still far from satisfactory. Current studies revealed that atrial fibrosis might promote recurrence of AF post-ablation (24), which indicates that atrial disease or cardiomyopathy plays an important role in AF maintenance and progression. Atrial fibrosis could lead to atrial structural and functional abnormality, behind which underlies various diseases. Hence, the concept of “atrial cardiomyopathy (ACM)” developed and the close relationship between ACM and AF has become a common knowledge (19, 25). The current predictors of AF mainly focused on fibrotic, structural, and atrial electrical indicators including BNP (brain natriuretic peptide), sST2 (soluble growth stimulator gene 2 protein) (26), cardiac magnetic resonance, LAD, LVEF, atrial volume, and others. However, all these indicators are directly or indirectly atrial disease or atrial fibrosis related. Therefore, searching for an easily acquired and more accessible predictor suggesting that atrial disease is more important and practical.

### Electrocardiogram Indicators and AF

Since AF is closely associated with atrial fibrosis of the left atrium, ECG indicators that could predict the occurrence of AF are usually related to the electrical activation of the left atrium. Previous studies found that prolonged P wave duration (20, 27), amplitude of P wave in lead II and V1 (28), and intra-atrial conduction time (21) were closely related to the occurrence of AF, which was consistent with the results of this study. During typical AFL, the activation of left atrium comes from the bottom of the left atrium going upward to the roof, which forms the negative component of the F wave in the inferior leads as shown in Figure 3. The prolonged duration of this component indicates delayed activation of left atrium. Left atrial disease can manifest with abnormalities in these two indicators and they can serve as predictors of AF occurrence post-CCW-AFL ablation besides the SR predictors.

This study presents evidence to show that ECG parameters both during the SR and CCW-AFL have predictive value in new-onset AF after CCW-AFL ablation. However, no significant difference was found between AF and non-AF groups with respect to left atrium diameter. The possible explanation might be that patients enrolled in this study were without documented AF, indicating a relatively early stage of left atrial disease with “structurally normal” left atrium. However, even an early stage of left atrial disease could result in abnormal left atrial electrical activation, which could be reflected on surface ECG. From this point of view, ECG indicators might have advantages in predicting occurrence of AF in the early stage of left atrial disease. Interestingly, we found that independent predictive values were all time duration-related indicators. However, parameters related to the amplitude had no independent predictive value according to the results of this study. We speculate that the structural remodeling of atrial disease includes hypertrophy and dilation. Hypertrophy usually manifests as an increased amplitude, whereas dilation usually manifests as a decreased amplitude and prolonged activation. Therefore, different types and stages of atrial disease might result in different changes in amplitude, which may weaken the predictive value of the amplitude. However, electrical activation of left atrium would be prolonged regardless of the types and stages of left atrial disease, resulting in a more important role of duration-dependent parameters in AF prediction. In addition, among those amplitude-dependent predictors, A_FNII_ had predictive value, whereas no significant differences were found between the two groups with respect to A_PNV1_ according to this study. The possible reasons are as follows: (1) small sample size; (2) the amplitude of the negative component in lead V1 was sometimes too small, leading to deviated measurement; and (3) when projecting on the surface ECG, activation during different rhythm has different blinding areas. Thus, the predictors during SR can be supplemented by AFL predictors.

### Clinical Implications

Combined ECG indicators during both the CCW-AFL and SR are the direct and easily accessible parameters for predicting newly developed AF post-AFL ablation. With this, those patients who are at high risk of AF occurrence could receive both the AFL ablation and PVI in the same procedure to avoid a second AF ablation or need to have a close follow-up to receive early AF management. Developing a scoring system to stratify the patients with CCW-AFL and to further verify its predictive value is very necessary.

### Study Limitations

First, this was a single-center study with a relatively small sample size. The predictive value of the ECG predictors needs to have further large-scale prospective studies. A predictive scoring system can be established and the positive predictive value can be verified. Second, despite regular follow-up, asymptomatic paroxysmal episodes of AF were difficult to exclude, leading to underestimation of the incidence of new-onset AF. As long-term event monitors such as a 30-day event monitors/Holter were unavailable, subclinical preexisting AF was difficult to exclude. Third, the incorporation of other clinical characteristics such as BNP and fibrosis assessment could make the scoring system more accurate. Fourth, as some ECGs collected a period before enrolment was measured, it was difficult to exclude the potential influence of AADs on ECG parameters under recall bias. Last but not least, MRI imaging, atrial refractory periods, and left atrial electroanatomical mapping were not performed to better verify the underlying mechanisms.

## Conclusion

Parameters representing left atrial activation time under both the SR and AFL were independently associated with new-onset AF post-typical AFL ablation and may be useful in risk prediction, which needs to be confirmed by further prospective studies.

## Data Availability Statement

The original contributions presented in the study are included in the article/Supplementary Material, further inquiries can be directed to the corresponding author/s.

## Ethics Statement

The studies involving human participants were reviewed and approved by Ethics Committee of the First Affiliated Hospital of Nanjing Medical University. The patients/participants provided their written informed consent to participate in this study. Written informed consent was obtained from the individual(s) for the publication of any potentially identifiable images or data included in this article.

## Author Contributions

All authors listed have made a substantial, direct and intellectual contribution to the work, and approved it for publication.

## Funding

This work was funded by Beijing Xinlian Zhicheng Cardiovascular Health Public Welfare Foundation (grant number 303103505CA20).

## Conflict of Interest

MC reports receiving lecture fees from Biosense Webster, Abbott, Medtronic, Boston Scientific, Bayer, and Boehringer Ingelheim. The remaining authors declare that the research was conducted in the absence of any commercial or financial relationships that could be construed as a potential conflict of interest.

## Publisher's Note

All claims expressed in this article are solely those of the authors and do not necessarily represent those of their affiliated organizations, or those of the publisher, the editors and the reviewers. Any product that may be evaluated in this article, or claim that may be made by its manufacturer, is not guaranteed or endorsed by the publisher.

## References

[1] Brembilla-Perrot B Girerd N Sellal JM Olivier A Manenti V Villemin T. Risk of atrial fibrillation after atrial flutter ablation: impact of AF history, gender, and antiarrhythmic drug medication. J Cardiovasc Electrophysiol. (2014) 25:813–20. 10.1111/jce.12413 24654647

[2] Chinitz JS Gerstenfeld EP Marchlinski FE Callans DJ. Atrial fibrillation is common after ablation of isolated atrial flutter during long-term follow-up. Heart Rhythm. (2007) 4:1029–33. 10.1016/j.hrthm.2007.04.002 17675077

[3] Seara JG Roubin SR Gude Sampedro F Barreiro VB Sande JM Manero MR. Risk of atrial fibrillation, stroke, and death after radiofrequency catheter ablation of typical atrial flutter. Clin Res Cardiol. (2014) 103:543–52. 10.1007/s00392-014-0682-6 24566731

[4] Tomson TT Kapa S Bala R Riley MP Lin D Epstein AE. Risk of stroke and atrial fibrillation after radiofrequency catheter ablation of typical atrial flutter. Heart Rhythm. (2012) 9:1779–84. 10.1016/j.hrthm.2012.07.013 22813577

[5] Andersen K Rasmussen F Neovius M Tynelius P Sundstrom J. Body size and risk of atrial fibrillation: a cohort study of 1.1 million young men. J Intern Med. (2018) 283:346–55. 10.1111/joim.12717 29178512

[6] Asad Z Abbas M Javed I Korantzopoulos P Stavrakis S. Obesity is associated with incident atrial fibrillation independent of gender: a meta-analysis. J Cardiovasc Electrophysiol. (2018) 29:725–32. 10.1111/jce.13458 29443438

[7] Aune D Feng T Schlesinger S Janszky I Norat T Riboli E. Diabetes mellitus, blood glucose and the risk of atrial fibrillation: a systematic review and meta-analysis of cohort studies. J Diabetes Complicat. (2018) 32:501–11. 10.1016/j.jdiacomp.2018.02.004 29653902

[8] Miyazawa K Kondo Y Nakano M Esteve-Pastor MA Rivera-Caravaca JM Senoo K. Risk factors for the development of incident atrial fibrillation in patients with cardiac implantable electronic devices. Eur J Intern Med. (2018) 52:54–9. 10.1016/j.ejim.2018.02.019 29490874

[9] Horio T Iwashima Y Kamide K Tokudome T Yoshihara F Nakamura S. Chronic kidney disease as an independent risk factor for new-onset atrial fibrillation in hypertensive patients. J Hypertens. (2010) 28:1738–44. 10.1097/HJH.0b013e32833a7dfe 20485194

[10] Liu F Xin Z Bin Waleed K Lin Y Tse G Luhanga A. CHA2DS2-VASc score as a predictor of new-onset atrial fibrillation after catheter ablation of typical atrial flutter. Front Physiol. (2020) 11:558. 10.3389/fphys.2020.00558 32587524 PMC7298125

[11] Lee YS Hyun DW Jung BC Cho YK Lee SH Shin DG. Left atrial volume index as a predictor for occurrence of atrial fibrillation after ablation of typical atrial flutter. J Cardiol. (2010) 56:348–53. 10.1016/j.jjcc.2010.07.006 20889311

[12] Enriquez A Sarrias A Villuendas R Ali FS Conde D Hopman WM. New-onset atrial fibrillation after cavotricuspid isthmus ablation: identification of advanced interatrial block is key. Europace. (2015) 17:1289–93. 10.1093/europace/euu379 25672984

[13] Tse G Lakhani I Zhou J Li KHC Lee S Liu Y. P-wave area predicts new onset atrial fibrillation in mitral stenosis: a machine learning approach. Front Bioeng Biotechnol. (2020) 8:479. 10.3389/fbioe.2020.00479 32500070 PMC7243705

[14] Ellis K Wazni O Marrouche N Martin D Gillinov M McCarthy P. Incidence of atrial fibrillation post-cavotricuspid isthmus ablation in patients with typical atrial flutter: left-atrial size as an independent predictor of atrial fibrillation recurrence. J Cardiovasc Electrophysiol. (2007) 18:799–802. 10.1111/j.1540-8167.2007.00885.x 17593230

[15] Sun GZ Guo L Wang XZ Song HJ Li Z Wang J. Prevalence of atrial fibrillation and its risk factors in rural China: a cross-sectional study. Int J Cardiol. (2015) 182:13–7. 10.1016/j.ijcard.2014.12.063 25576710

[16] Sasaki K Sasaki S Kimura M Owada S Horiuchi D Itoh T. Revisit of typical counterclockwise atrial flutter wave in the ECG: electroanatomic studies on the determinants of the morphology. Pacing Clin Electrophysiol. (2013) 36:978–87. 10.1111/pace.12129 23594189

[17] Rodriguez LM Timmermans C Nabar A Hofstra L Wellens HJ. Biatrial activation in isthmus-dependent atrial flutter. Circulation. (2001) 104:2545–50. 10.1161/hc4601.097996 11714648

[18] Tai CT Chen SA. Electrophysiological mechanisms of atrial flutter. J Chin Med Assoc. (2009) 72:60–7. 10.1016/S1726-4901(09)70024-3 19251532

[19] Guichard JB Nattel S. Atrial cardiomyopathy: a useful notion in cardiac disease management or a passing fad? J Am Coll Cardiol. (2017) 70:756–65. 10.1016/j.jacc.2017.06.033 28774383

[20] Muller-Edenborn B Minners J Kocher S Chen J Zeh W Lehrmann H. Amplified P-wave duration predicts new-onset atrial fibrillation in patients with heart failure with preserved ejection fraction. Clin Res Cardiol. (2020) 109:978–87. 10.1007/s00392-019-01590-z 31863175

[21] Alexander B Milden J Hazim B Haseeb S Bayes-Genis A Elosua R. New electrocardiographic score for the prediction of atrial fibrillation: the MVP ECG risk score (morphology-voltage-P-wave duration). Ann Noninvasive Electrocardiol. (2019) 24:e12669. 10.1111/anec.12669 31184409 PMC6931412

[22] Martinez A Alcaraz R Rieta JJ. Study on the P-wave feature time course as early predictors of paroxysmal atrial fibrillation. Physiol Meas. (2012) 33:1959–74. 10.1088/0967-3334/33/12/1959 23138002

[23] Haissaguerre M Jais P Shah DC Takahashi A Hocini M Quiniou G. Spontaneous initiation of atrial fibrillation by ectopic beats originating in the pulmonary veins. N Engl J Med. (1998) 339:659–66. 10.1056/NEJM199809033391003 9725923

[24] den Uijl DW Cabanelas N Benito EM Figueras R Alarcon F Borras R. Impact of left atrial volume, sphericity, and fibrosis on the outcome of catheter ablation for atrial fibrillation. J Cardiovasc Electrophysiol. (2018) 29:740–6. 10.1111/jce.13482 29528532

[25] Bisbal F Baranchuk A Braunwald E Bayes de Luna A Bayes-Genis A. Atrial failure as a clinical entity: JACC review topic of the week. J Am Coll Cardiol. (2020) 75:222–32. 10.1016/j.jacc.2019.11.013 31948652

[26] Liu H Wang K Lin Y Liang X Zhao S Li M Chen M. Role of sST2 in predicting recurrence of atrial fibrillation after radiofrequency catheter ablation. Pacing Clin Electrophysiol. (2020) 43:1235–41. 10.1111/pace.14029 32735032

[27] Caldwell J Koppikar S Barake W Redfearn D Michael K Simpson C. Prolonged P-wave duration is associated with atrial fibrillation recurrence after successful pulmonary vein isolation for paroxysmal atrial fibrillation. J Interv Card Electrophysiol. (2014) 39:131–8. 10.1007/s10840-013-9851-1 24306110

[28] Yoshizawa TNS Niwano H Igarashi T Fujiishi T Ishizue N Oikawa J. Prediction of new onset atrial fibrillation through P wave analysis in 12 lead ECG. Int Heart J. (2014) 55:422–7. 10.1536/ihj.14-052 25098176

